# Nurse's Perceptions and Roles in the Management of Chronic Kidney Disease‐Associated Pruritus: A Multicentre Survey Across Europe

**DOI:** 10.1111/jorc.70018

**Published:** 2025-05-13

**Authors:** Anastasia Liossatou, Afra Masià‐Plana, Veronica Strini, Davide Sisti, Ilaria de Barbieri, Sofia Zyga

**Affiliations:** ^1^ Dialysis Unit The General Hospital of Kefalonia Argostoli Kefalonia Greece; ^2^ Department of Nursing, Faculty of Health Sciences University of Peloponnese Greece; ^3^ Department of Nursing University of Girona Girona Spain; ^4^ Clinical Research Unit‐Azienda Ospedale Università Padova Padova Italy; ^5^ Department of Biomolecular Sciences, Service of Biostatistics University of Urbino Urbino Italy; ^6^ Healthcare Professionals Department Azienda Ospedale Università di Padova Padova Italy

**Keywords:** chronic kidney disease associated pruritus, haemodialysis, itching, nurses’ perceptions, nurses’ roles

## Abstract

**Background:**

Chronic kidney disease‐associated pruritus is a debilitating condition affecting people on haemodialysis, and nephrology nurses have the potential to play a crucial role in its early identification and management.

**Objectives:**

To investigate the nephrology nurses’ perceptions and roles in the identification and management of chronic kidney disease‐associated pruritus among people receiving haemodialysis.

**Design:**

A survey‐based, cross‐sectional study was conducted by the European Dialysis Transplant Nurses Association/European Renal Care Association, using a structured questionnaire designed by the researchers and distributed online.

**Participants:**

Nephrology nurses working in the haemodialysis field.

**Results:**

A total of 286 questionnaire submissions were received from 15 European countries. The study sample comprised 48 male and 238 female nurses. The findings reveal that 71.9% of nurses participating in the study widely agreed that the people on haemodialysis under their care tend to withhold reporting symptoms of chronic kidney disease‐associated pruritus. Nurses perceived that approximately 25% of their people on haemodialysis did not openly discuss their symptoms of pruritus with their care team. Most nurses (76.4%) reported being involved in recommending or prescribing treatments for chronic kidney disease‐associated pruritus, with 71.4% specifically addressing treatments for itching. However, 35.5% of healthcare professionals do not fully recognise the link between pruritus and kidney disease.

**Conclusion:**

The study highlights the complex challenges nephrology nurses face in identifying and managing chronic kidney disease‐associated pruritus. It emphasises the significant impact of pruritus on people's quality of life and the crucial role nurses can play in early detection and management.

## 1 Introduction

Pruritus, or itch, is the unpleasant sensation of the skin that provokes the desire for scratching. It can be acute or chronic, with the latter being defined as itch that lasts for at least 6 weeks. Several conditions may be responsible for the induction of chronic pruritus, such as dermatologic, systemic, neurologic, or psychogenic diseases, alone or combined (Bathe et al. 2009; Ständer et al. 2007; Weisshaar, Szepietowski, et al. 2019).

Chronic kidney disease (CKD) is an example of a systemic disease that causes itch (Ständer et al. 2007; Weisshaar, Szepietowski, et al. 2019). CKD‐associated Pruritus (CKD‐aP) is a debilitating condition, characterised by moderate‐to‐severe itch, affecting people with end‐stage kidney disease undergoing haemodialysis (HD) (Mathur et al. 2010). However, CKD‐aP is not limited to people undergoing HD and is equally common among those with non‐dialysis CKD (Sukul et al. 2019) as well as the individuals on peritoneal dialysis (PD) (Tennankore et al. 2024). For the purposes of this manuscript and survey, the content will particularly focus on individuals experiencing CKD‐associated pruritus who are undergoing HD.

## 2 Literature Review

### 2.1 Signs and Symptoms of CKD‐aP

The clinical presentation of CKD‐aP is variable, and no specific profile can characterise a typical patient. Since itching has a range of severity, it can be sporadic or continuous and it may occur anytime during the day (Rayner et al. 2017; Shirazian et al. 2017; Verduzco and Shirazian 2020). The skin of affected people may often be unchanged (being dry and scaly like that of unaffected people), or present with skin changes secondary to intense scratching activity (excoriations with and without impetigo, crusts, papules, ulcerations, erosions and less commonly prurigo nodularis). CKD‐aP may affect specific skin areas, mainly the back, face and shunt arm, or it can be generalised, and often symmetrical (Manenti and Leuci 2021; Mettang and Kremer 2015; Verduzco and Shirazian 2020).

### 2.2 Prevalence and Burden of CKD‐aP

Data from the Dialysis Outcomes and Practice Patterns Study (DOPPS), phases 4–6 (2009–2018), indicated that 67% of individuals on HD reported being affected by CKD‐aP, with 37% experiencing moderate‐to‐severe symptoms of CKD‐aP (Sukul et al. 2021). CKD‐aP is also common among people with non‐dialysis CKD. It has been found that 24% of people across all stages of CKD suffer from at least moderate pruritus, while the prevalence of severe‐to‐extreme pruritus ranges from 10% to 13% (Sukul et al. 2019). Additionally, according to a recent study, it was identified that 43% of individuals on PD had moderate‐to‐extreme pruritus and it was associated with poor health outcomes and mortality or transfer to HD (Tennankore et al. 2024).

Despite its high prevalence, CKD‐aP remains underreported by people and underestimated by healthcare professionals, leading to its underdiagnosis and undertreatment (Rayner et al. 2017; Sukul et al. 2021). As a result, unmanaged CKD‐aP profoundly affects people's quality of life with a dreadful impact on sleep quality, mental health and social well‐being, whilst it is also associated with poor clinical outcomes (Mathur et al. 2010; Narita et al. 2006; Pisoni et al. 2006; Sukul et al. 2021).

People bothered by itchy skin also report that CKD‐aP influences their social interactions, either sometimes or always (Rayner et al. 2017). Apart from social functioning, CKD‐aP has also an impact on the ability of people to work, and is associated with lower rates of employment (Rayner et al. 2017; Sukul et al. 2021). Decreased employment prevalence among people who are extremely bothered by pruritus may be attributed to symptoms burden, which makes it difficult for them to either seek or maintain a job position (Sukul et al. 2021).

In addition, a DOPPS analysis including 23,264 people on HD from 21 countries showed that adjusted rates of all‐cause hospitalisations are greater for people moderately‐to‐severely versus people not at all bothered by itchy skin (Sukul et al. 2021). Consequently, utilisation of hospital resources, such as erythropoietin‐stimulating agents, intravenous iron and antibiotics increases. Moreover, the number of missed dialysis sessions increases with itching severity (Sood et al. 2013), while in most severe cases, CKD‐aP has been found to be an independent predictive factor for mortality (Narita et al. 2006; Sukul et al. 2021).

### 2.3 Identification and Measurement of CKD‐aP

Diagnosis of CKD‐aP depends on people reporting their symptoms. Thus, it is very important for healthcare professionals in kidney care to frequently enquire whether individuals with CKD experience itching (Agarwal et al. 2022; Manenti and Leuci 2021).

People with CKD usually have dry and atrophic skin, with skin xerosis possibly acting as a co‐factor in promoting CKD‐aP. Clinical examination of the patient, including a thorough inspection of the entire skin, is an important part of the diagnostic procedure to exclude the presence of other dermatological diseases (Manenti and Leuci 2021; Weisshaar, Matterne, et al. 2019). Laboratory screening, including, hepatic serology, thyroid‐stimulating hormone, ferritin, glucose, C‐reactive protein, dialysis efficiency (Kt/V) and calcium‐phosphorus product, is also recommended to detect other underlying diseases responsible for chronic CKD‐aP (Manenti and Leuci 2021).

Several simple self‐reported assessment scales are available to evaluate the presence and severity of CKD‐aP (Manenti and Leuci 2021; Verduzco and Shirazian 2020). Currently used scales can be divided into three categories:

- severity scales that measure itch severity, such as the Visual Analogue Scale (VAS), the Numeric Rating Scale (NRS), the Verbal Rating Scale (VRS) and one question from the Kidney Disease QoL‐Short Form (KDQOL‐SF).
- multidimensional scales which measure several itch characteristics, such as the 5‐D itch scale and the itching severity scale (ISS).
- quality of life scales assessing the impact of itching on people's quality of life, such as the Skindex‐10, the Itch Medical Outcomes Study (itch MOS) and the Self‐Assessed Disease Severity (SADS).

According to the proposed algorithm of Agarwal et al. (2022), in case CKD‐aP is diagnosed, the nephrology care team is further suggested to assess its intensity, as well as its effect on people’ quality of life using patient‐ reported outcome (PRO) scales, such as WI‐NRS and SADS (Agarwal et al. 2022).

### 2.4 The Role of Nurses in the Identification and Management of People With CKD‐aP

It has been found that people are most likely to discuss their condition with nephrology nurses, along with nephrologists (Mohamed et al. 2020; Rayner et al. 2017). Nurses may play an important role in the identification and severity assessment of individuals with CKD‐aP, due to their increased face‐to‐face interaction time (Mohamed et al. 2020). People with CKD‐aP may hesitate to disclose symptoms of itching, considering them to be a minor issue or a normal feature of their condition. Nurses are in a unique position to detect signs of CKD‐aP during routine interactions, assessments, and discussions with people under their care.

Nurses can significantly impact the lives of people by proactively addressing CKD‐aP. Recognising the condition, facilitating the implementation of appropriate treatments, such as lifestyle modifications or pharmacological interventions, and offering education on symptom management are all components of this. Additionally, nurses may advocate for the inclusion of CKD‐aP management in a comprehensive care plan to prevent it from being disregarded.

Furthermore, nurses can encourage people to actively participate in the decision‐making of their treatment, to ensure optimal management (Rhee et al. 2022). Nurses may have a primary responsibility in reinforcing the importance of compliance in the treatment and providing the multidisciplinary approach people need to manage CKD‐aP (Mohamed et al. 2020). It is essential to resolve CKD‐aP to enhance people's quality of life and outcomes. The attentiveness and intervention of nurses can alleviate unnecessary suffering and assist people in living with greater comfort and dignity throughout their CKD journey.

However, clinical practice guidelines with regard to CKD‐aP need to be developed so that healthcare professionals have sufficient evidence on the available treatment options (Aresi et al. 2019).

### 2.5 Aim of the Study

There is no research conducted on the perceptions or knowledge of nurses in relation to CKD‐aP as well as the roles nurses assume in the identification and management of CKD‐aP. among people receiving HD. Therefore, the study aimed to investigate the nephrology nurses’ perceptions and roles in the identification and management of people with CKD‐aP receiving HD.

## 3 Materials and Methods

A survey‐based, cross‐sectional study was conducted by the European Dialysis Transplant Nurses Association/European Renal Care Association (EDTNA/ERCA), using an Ad Hoc structured questionnaire that was designed by a team of nurses and distributed online via the Google Forms platform to reach EDTNA/ERCA members in Europe during the period of 15 May– 15 August 2023. This survey was originally developed in English and then it was translated into 10 languages by nurses who are native speakers and members of EDTNA/ERCA: Croatian, Dutch, French, German, Greek, Polish, Portuguese, Spanish, Swedish, and Turkish.

The questionnaire was addressed to approximately 550 EDTNA/ERCA members, via a survey link distributed through email using EDTNA/ERCA's extensive membership database. Relevant content was included in the accompanying letter. The inclusion criteria encompassed nephrology nurses of all levels employed in the HD field of kidney care with at least 1 year of experience. The questionnaire of 41 (items) closed‐ended multiple‐choice questions sought to investigate the following aspects (Appendix S1):

1. The socio‐demographic characteristics of the nephrology nurses.
2. The percentage of people they attend with officially diagnosed CKD‐aP.
3. The characteristics of HD treatment in people with CKD‐aP.
4. The interventions followed by nurses during a typical HD treatment in people with CKD‐aP.
5. Whether people with CKD‐aP are reporting/talking about their itching with their nurses or other colleagues.
6. What challenges people with CKD‐aP are sharing/discussing with their nurses.
7. The reasons why people with CKD‐aP do not report/talk about their symptoms.
8. The nurses’ roles in the identification and management of people with CKD‐aP and their interacting within the multidisciplinary team.
9. The way people manage/treat their pruritus.
10. The level of knowledge about available treatment for CKD‐aP, and
11. Whether burden of CKD‐aP is of concern to the caregivers/people's families.

The study received a favourable acceptance from the Ethics Committee.

### 3.1 Statistical Analysis

The study adopted a quantitative research methodology with descriptive statistics. Sociodemographic characteristics of participants, namely age and years of experience, were summarised using means and standard deviations (SD). The multiple‐choice survey data were analysed using descriptive statistics. The results were presented as percentages showing the distribution of perceptions among the study population. The analysis was performed using SPSS 23 and Excel. No inferential statistics were used as the primary aim was to describe trends and distributions within the data.

## 4 Results

A total of 286 questionnaire submissions were received from nurses working in the HD field of kidney care across 15 European countries, yielding a response rate of 52%. The study sample comprised 48 male and 238 female nurses. Their characteristics are presented in Table 1. According to respondents, there was no significant age difference between male and female nurses (*t* = 0.86, *p* = 0.39). A marginally significant difference was reported in years of general nursing experience (*t* = 1.85, *p* = 0.07), with female nurses indicating slightly more experience on average. However, no significant gender difference was perceived in years working specifically in nephrology nursing (*t* = 0.19, *p* = 0.85), suggesting comparable experience in this specialty. In terms of educational background, most respondents reported holding a Bachelor's degree (54.2% of males; 67.6% of females), followed by a Master's degree (41.7% of males; 28.2% of females), while only 4.2% of both male and female nurses reported having a PhD. The majority identified as Specialist Nurses, with males more frequently reporting roles as Head Nurse/Nursing Manager, and females more commonly identifying as Staff Nurses (Table 1).

**Table 1:** Study sample characteristics (*N* = 286).

	Male	Female	*t* (prob)
Age, mean ± SD	42.9 ± 10.8	44.4 ± 10.7	0.86 (0.39)
Years of working in nursing, mean ± SD	15.9 ± 12.6	19.5 ± 11.4	1.85 (0.07)
Years of working in nephrology nursing, mean ± SD	13.9 ± 12.0	13.5 ± 12.4	0.19 (0.85)
			***χ*** ^**2**^ **(prob)**
Educational level, *N*			
PhD Degree	2 (4.2)	10 (4.2)	3.51 (0.17)
Master Degree	20 (41.7)	67 (28.2)	
Bachelor Degree	26 (54.2)	161 (67.6)	
Position at work, *N*			
Head Nurse/Nursing Manager	18 (37.5)	64 (27)	2.71 (0.26)
Specialist Nurse	21 (43.75)	108 (45.6)	
Staff Nurse	9 (18.75)	65 (27.4)	

Abbreviation: SD, standard deviation.

### 4.1 Prevalence and Incidence of CKD‐aP

Nurses’ perceptions regarding the prevalence of chronic kidney disease‐associated pruritus (CKD‐aP) among people on HD varied. Most nurses (26% of the total respondents) estimated that 0%–5% of people under their care experienced CKD‐aP. Additionally, 37% believed that 6%–20% of individuals had received a diagnosis of CKD‐aP (Item 9, Table 2).

**Table 2:** Prevalence, incidence, treatments and practices for the management of patients with CKD‐associated pruritus.

Items		*N*	%
9—How many patients have officially been diagnosed with CKD‐associated Pruritus (uremic pruritus, chronic itching)?	0%–5%	165	39.3
	6%–20%	155	36.9
	21%–30%	35	8.3
	31%–40%	25	6.0
	41%–50%	28	6.7
	Over 50%	12	2.9
10—Of those diagnosed with CKD‐associated Pruritus, how many are mentioning/talking about their itching with you or your colleagues?	None	38	9.0
	0%–5%	82	19.5
	6%–20%	105	25.0
	21%–30%	65	15.5
	31%–40%	0	0.0
	41%–50%	63	15.0
	Over 50%	67	16.0
11—Of those diagnosed with CKD‐associated Pruritus, what is their vascular access type:	A‐V Fistula	211	50.2
	A‐V Graft	83	19.8
	Central Venous Catheter	126	30.0
12—Of those diagnosed with CKD‐associated Pruritus, what is the method of their treatment:	HD	169	40.2
	OLHDF	186	44.3
	Home Nocturnal HD/OLHDF	65	15.5
13—Of those diagnosed with CKD‐associated Pruritus, what is the dialyser membrane type:	Low flux	115	27.4
	High Flux	256	61.0
	High Cut‐off	49	11.7
14—During the typical dialysis procedure, from the priming of blood lines and dialyser until the moment the patient connects to and eventually disconnects from the dialysis session, are there any different nursing interventions you follow or additional steps that you take with the patients with CKD‐associated Pruritus?	Yes	180	42.9
	No	240	57.1
15—If yes, what are they?	Special priming process‐ flushing with extra saline bloodlines and dialyser	52	12.4
	Use of special dialyser	61	14.5
	Administer tablets or IV antihistamine medication	43	10.2
	Administer tablets or IV corticosteroid medications	12	2.9
	Apply creams	12	2.9
	I answered no	240	57.1
16—Do you think patients do not report CKD‐associated Pruritus symptoms?	Yes	302	71.9
	No	118	28.1
17—If yes, why?	They don't feel comfortable to talk about it	77	25.5
	They feel embarrassed in case you see how bad their scratches are	58	19.2
	They feel it is less important	45	14.9
	They feel there is no available treatment for their problem	46	15.2
	They don't make the association between the symptom and the condition	76	25.2
18—Do you ever prescribe yourself or recommend to the nephrologist and/or patient any treatments overall?	Yes	321	76.4
	No	99	23.6
19—Do you ever prescribe yourself or recommend to the nephrologist and/or patient any treatment for itchiness specifically?	Yes	300	71.4
	No	120	28.6

Abbreviations: A‐V, arteriovenous; CKD, chronic kidney disease; HD, haemodialysis; IV, intravenous; OLHDF, online haemodiafiltration.

Despite this, nurses perceived that approximately 25% of the people under their care on HD did not openly discuss their symptoms of pruritus with their healthcare team. Only 16.0% of nurses reported individuals extensively discussed their symptoms (Item 10, Table 2), indicating a potential communication gap in symptom reporting.

### 4.2 Treatments and Practices

When asked about treatment approaches, nurses most frequently identified online haemodiafiltration (44.3%) and standard HD (40.2%) (item 12) as commonly used for managing CKD‐aP. Regarding vascular access, nurses reported that 50.2%) of their people received treatment through anA‐V fistula (whereas 61.0% of them utilised high‐flux dialyser membranes as the standard practice (Items 11–13, Table 2).

In terms of procedural practices during dialysis sessions for individuals with CKD‐aP, 57.1% of nurses indicated that no additional or specific interventions were typically implemented. Among those who did report additional measures, the most commonly cited practices included special priming processes (12.4%), use of special dialysers (14.5%), administration of antihistamines (10.2%), corticosteroids (2.9%), application of creams (2.9%), and other unspecified practices (17.6%) (Items 14 and 15, Table 2).

Nurses widely agreed (71.9%) that people on HD tend to withhold reporting symptoms of CKD‐aP. The perceived reasons for this included discomfort in discussing symptoms (25.5%), embarrassment about the appearance of scratched skin (19.2%), considering the issue to be minor (14.9%), believing no treatment options are available (15.2%), and not recognising the link between the symptom and their condition (25.2%) (Items 16 and 17, Table 2).

### 4.3 Healthcare Professionals' Perspectives and Patient Education

Most nurses (76.4%) reported being involved in recommending or prescribing treatments for CKD‐aP, with 71.4% specifically addressing treatments for itching (Items 18 and 19, Table 2). However, 35.5% of respondents indicated that healthcare professionals do not fully acknowledge the association between pruritus and CKD, even if they recognise its clinical relevance (Item 24, Table 3).

**Table 3:** Healthcare professionals’ perspectives, patient education, interprofessional communication and collaboration for the management of patients with CKD‐associated pruritus.

Items		*N*	%
20—Are there any patients in your centre who are not officially diagnosed with pruritus and yet present symptoms of CKD‐associated Pruritus?	Yes	354	84.3
	No	66	15.7
21—If yes, can you estimate approximately what proportion of all your patients does that represent?	0%–5%	91	25.7
	6%–20%	84	23.7
	21%–30%	67	18.9
	31%–40%	46	13.0
	41–50%	51	14.4
	over 50%	15	4.2
22—In case you detect potential symptoms (namely itching) of CKD‐associated Pruritus, do you document them in patient's file?	Yes	301	71.7
	No	119	28.3
23—In case you detect potential symptoms (namely itching) of CKD‐associated Pruritus, who do you report them to (please tick all that applies)?	Nephrologist	233	55.5
	Other nurse colleagues	124	29.5
	Dermatologist	25	6.0
	Psychologist	38	9.0
	Family member	0	0.0
24—Do you think that itching is considered by the nurses and other healthcare professionals as not linked to CKD?	Yes	149	35.5
	No	271	64.5
25—Do you usually encourage the patient to discuss about the itching problems with other health professionals within the nephrology care team?	Yes	331	78.8
	No	89	21.2
26—If yes, please specify who:	Nephrologist	142	42.9
	Other nurses	91	27.5
	Dermatologist	44	13.3
	Psychologist	27	8.2
	Family member	27	8.2
27—Do you usually discuss a patient's symptoms severity on CKD‐associated Pruritus with the nephrologist?	Yes	335	79.8
	No	85	20.2
28—If yes, what do you discuss about?	The impact CKD‐associated Pruritus has on patient's life	96	28.7
	Possible medication options	75	22.4
	The therapeutic results of medication administered during an HD session or medication taken at home	105	31.3
	Other	59	17.6
29—Do you take part in the decision‐making process with regard to?	Increase/decrease the frequencies of dialysis session?	108	25.7
	Increase/decrease in their duration?	86	20.5
	Adjustments to the dialysis parameters	121	28.8
	No, I am not involved in any way in the decision‐making process for any of the above	105	25.0
30—Do you ever discuss or interact with healthcare professionals other than the nephrologist with regard to a patient with CKD‐associated Pruritus?	Yes	245	58.3
	No	175	41.7
31—If yes, with who (please tick all that applies)?	Dermatologist	89	36.3
	Dietician	72	29.4
	Pharmacist	20	8.2
	Psychologist	19	7.8
	Family doctor	45	18.4

Abbreviations: CKD, chronic kidney disease; HD, haemodialysis.

A large proportion of nurses (84.3%) reported having observed symptoms consistent with CKD‐aP in individuals who did not have an official diagnosis. However, estimates of the prevalence of such undiagnosed cases varied widely across respondents, from 0% to 30%, possibly reflecting differing levels of clinical exposure or awareness (Items 20 and 21, Table 3).

Nurses generally promote discussions regarding CKD‐aP with the nephrologist (as reported by 80% of respondents; Item 27), which may indicate an ongoing effort to address symptom management, despite continuing gaps in understanding the condition's association with CKD.

### 4.4 Interprofessional Communication and Collaboration

Most nurses (79.8%) reported engaging in discussions with nephrologists about the severity of the people they experience CKD‐aP symptoms. According to the nurses, these conversations typically focus on the impact of symptoms on people's lives (28.7%), available medication options (22.4%), and observed therapeutic effects of treatments either administered during dialysis or taken at home (31.3%) (Items 27 and 28, Table 3).

Respondents also indicated active participation in dialysis‐related decision‐making. Specifically, 25.7% reported being involved in decisions about session frequency, 20.5% in modifying session duration, and 28.8% in adjusting dialysis parameters (Items 29–31, Table 3).

### 4.5 Challenges and Patient Self‐Medication

According to nurses, people with CKD‐aP face a range of challenges that negatively impact quality of life. These include sleep disturbances, social withdrawal, and the need to take additional medications (Items 32 and 33, Table 4).

**Table 4:** Challenges and patient self‐medication, nurses’ knowledge of the treatment options and involvement of family members for the management of patients with CKD‐associated pruritus.

Items		*N*	%
32—Do patients talk about the challenges that CKD‐associated Pruritus can pose in the day‐to‐day life (chronic itching) with you?	Yes	281	66.9
	No	139	33.1
33—If yes, what are these challenges (please tick all that applies)?	Sleep disorders	75	26.7
	Social isolation	38	13.5
	Difficulties in physical/professional activities	34	12.1
	Necessity to take additional medication to deal with itching or with new undesirable effects	38	13.5
	Changes in their diet since the start of itching or other undesirable effects linked to it	19	6.8
	Their feeling of embarrassment	32	11.4
	Their feeling of depression	27	9.6
	Sexual health issues	18	6.4
34—Do you think patients don't discuss CKD‐associated Pruritus symptoms with their doctors?	Yes	272	64.8
	No	148	35.2
35—If yes, why?	They don't feel comfortable to talk about it	68	25.0
	They feel embarrassed in case you see how bad their scratches are	34	12.5
	They feel it is less important	58	21.3
	They feel they will not get nursing/medical attention on this issue	56	20.6
	They don't relate itchiness to CKD	56	20.6
36—Do patients try self‐medication for their CKD‐associated Pruritus?	Yes	329	78.3
	No	91	21.7
37—If yes, what do the try (please tick all that applies)?	OTC creams	118	35.9
	Oils and herbs	113	34.3
	Antihistamine tablets	98	29.8
38—How do they come up with those remedies?	Internet	120	28.6
	Family member	109	26.0
	Treating specialist	58	13.8
	Suggested by another patient	77	18.3
	I don't know	56	13.3
39—Are there any treatments specifically for the itching that you know of available for the patients with CKD‐associated Pruritus (please tick all that applies)?	Gabapentin/pregabalin	85	20.2
	Antihistamines tablets	92	21.9
	Antihistamines creams	54	12.9
	Corticosteroids tablets	71	16.9
	Corticosteroids creams	65	15.5
	UVB (Ultraviolet phototherapy)	25	6.0
	No available treatment	65	15.5
40—Do relatives/caregivers mention/discuss itchiness with you?	Yes	226	53.8
	No	194	46.2
41—If yes, is itching a major topic of concern for the family?	Yes	125	55.3
	No	101	44.7

Abbreviations: CKD, chronic kidney disease; OTC, over‐the‐counter; UVB, ultraviolet B.

A majority of nurses (78.3%) believed that people under their care attempt to manage their symptoms through self‐medication. Among these, 35.9% were perceived to use over‐the‐counter products, while others turned to alternative treatments found online or suggested by family. In contrast, 21.7% of nurses reported that people under their care did not engage in self‐medication (Items 36–38, Table 4).

### 4.6 Nurses' Knowledge of Treatment Options and Family Involvement

When asked about their familiarity with pharmacological options for CKD‐aP, 21.9% of nurses reported awareness of antihistamine tablets, and 20.2% cited gabapentin or pregabalin as commonly used treatments (Items 39–41, Table 4).

Regarding family and caregiver involvement, nearly half of the nurses (46.2%) perceived that family members rarely mention or discuss itching symptoms. Even when the topic was raised, 44.7% of nurses believed it was not a major concern within the family (Items 39–41, Table 4).

## 5 Discussion

The novelty of this study lies in the first‐time investigation of nephrology nurses’ perceptions about CKD‐aP, as well as the roles they assume in its identification and management. The findings provide insights into the current state of the condition from the perspective of frontline healthcare professionals.

Regarding the aim of investigating perceptions of CKD‐aP, are consistent with existing literature (Rayner et al. 2017; Sukul et al. 2021), nurses reported that a high number of people undergoing HD do not openly discuss their symptoms of CKD‐aP with healthcare professionals. In fact, nurses perceived that only 16.0% of the people they care of, extensively discuss their symptoms, suggesting that those who do may represent a subset with more severe symptomatology or a greater willingness to communicate.

When reporting on the type of HD modality, individuals with CKD‐aP are receiving, nurses indicated that 40.2% of people were on conventional HD, while 44.3% were receiving OLHDF. It is important to note that these treatment modalities are not mutually exclusive, and individuals might be receiving both at different times.

In terms of nurses’ roles in identification and management, 78.8% of nurses reported that healthcare professionals actively support discussions regarding itching issues within the nephrology care team. This active involvement, combined with collaborative decision‐making processes that include other specialties such as dermatology, dietetics, and psychology, reflects a holistic approach to managing CKD‐aP and acknowledges its multifaceted nature (Cheng and Wong 2022). Additionally, nephrology nurses often take the initiative in prescribing or recommending treatments for CKD‐aP, particularly for managing itchiness.

Nurses also reported that 78.3% of individuals attempt self‐medication to alleviate their symptoms, predominantly using over‐the‐counter medications or alternative therapies. However, they observed that 21.7% of the people they care of do not engage in self‐medication, highlighting the need for tailored interventions that consider individual coping strategies and preferences.

Finally, while a variety of treatment options is available to healthcare professionals, nurses reported greater familiarity with pharmaceutical agents such as gabapentin/pregabalin and antihistamine tablets. The limited involvement of family members and caregivers in discussing symptoms with healthcare professionals suggests that there may be opportunities to enhance caregiver education and engagement in supporting people with CKD‐aP.

## 6 Study Limitation

This study has several limitations.

First, the survey was conducted exclusively in European countries, which restricts the generalisability of the findings on a global scale.

Second, the survey is inherently descriptive, as it collected observational data rather than utilising a questionnaire with validated scoring metrics, and the instrument itself has not undergone validation.

Third, the cross‐sectional design precludes any inference of causality. Furthermore, the analysis did not explore differences across countries or between public and private hospitals, nor did it stratify the results by age, sex, hospital role, educational qualification, or years of experience.

Lastly, the questionnaire did not include free‐text options; consequently, qualitative data were not collected. Although free‐text responses might have enriched the study design, the challenge of analysing extensive qualitative data is acknowledged. Instead, the survey exclusively captured quantitative data that were analysed descriptively.

## 7 Implications For Clinical Practice

Nurses have an active role in dialysis therapy and symptom management, and they may function as the key communication channel within the nephrology care team. Nurses are in a unique position to identify strategies in clinical practice and lead the management of the burdensome symptoms of CKD‐aP due to their close proximity to the patient and the increased likelihood of people sharing their symptom burden with them. (Mohamed et al. 2020; Rayner et al. 2017).

The findings highlight the need to increase healthcare professionals’ awareness of the association between pruritus and chronic kidney disease (CKD). Despite frequent observations of symptoms, a significant portion does not fully acknowledge this link, suggesting the need for targeted education. Moreover, systematically incorporating pruritus into nephrology team discussions could enhance early diagnosis and treatment, leading to more effective patient management.

## 8 Conclusion

The study provides insights into the current practices and perceptions of nephrology nurses regarding the identification and management of CKD‐aP. These insights point the complexity of current situation and the importance of addressing clinical, psychosocial, and educational aspects to optimise people’ outcomes and quality of life.

CKD‐aP has a major impact on people’ health‐related quality of life (Pisoni et al. 2006; Rayner et al. 2017; Sukul et al. 2021). Nurses have the potential to play a crucial role in its early identification and management, as well as encouragement of people to talk about their symptom, rather than suffering in silence.

The implications from possible future research may involve conducting studies aimed at developing and evaluating educational interventions for nephrology nurses. The objective would be to enhance their knowledge and abilities in the identification and management of people with CKD‐aP. In addition, future research might prioritise the assessment of the efficacy of nurse‐led interventions in managing the clinical, psychological, and educational elements of CKD‐aP, and their influence on people's outcomes and quality of life. Additionally, investigating the obstacles and factors that promote efficient communication between nurses and CKD people addressing CKD‐aP could be a valuable subject for future research.

## Author Contributions

Study conception and design: Anastasia Liossatou, Ilaria de Barbieri, Sofia Zyga. Data collection: Veronica Strini. Analysis and interpretation of results: Veronica Strini, Davide Sisti. Draft manuscript preparation: Anastasia Liossatou, Veronica Strini, Afra Masià‐Plana. All authors reviewed the results and approved the final version of the manuscript.

## Ethics Statement

The study Ethical approval was waived by the Ethical & Research Committee of the University of Peloponnese, Greece, with the protocol number Reg 9984/09‐05‐2023.

## Conflicts of Interest

The authors declare no conflicts of interest.

## Supporting information

Appendix.docx.
