# Supplementary material for: Nurse's Perceptions and Roles in the Management of Chronic Kidney Disease‐Associated Pruritus: A Multicentre Survey Across Europe
Source: J Ren Care. 2025 May 13;51(2):e70018. doi: 10.1111/jorc.70018 (PMC12096264; doi:10.1111/jorc.70018)
Supplement: Supplementary file 1 — Appendix.docx. [file JORC-51-0-s001.docx]

**Appendix**

**Survey on the perceptions and roles on the management of associated Pruritus among nephrology nurses of people with chronic kidney disease undergoing haemodialysis.**

Dear Colleagues,

I would like to invite you to fill in the following survey questionnaire on **chronic kidney disease-associated Pruritus (CKD -associated Pruritus)** also called Uremic Pruritus, and often referred to as itch/itching/itchiness by patients. This survey is being conducted by EDTNA/ERCA with the aim to explore the perceptions and roles of nephrology nurses on the management of patients suffering from CKD-associated Pruritus. There is no right or wrong answer. You have the right to refuse to answer questions or withdraw at any time. Any information you provide to us will be treated in the strictest confidence.

**Abbreviations used**

CKD = chronic kidney disease

HD = haemodialysis

OLHDF= online haemodialfitration

OTC: over-the-counter

**Data privacy**

Participants in this anonymous survey take note and acknowledge by ticking the box below that any personal data if collected by EDTNA/ERCA or submitted by the participant and managed by or for EDTNA/ERCA is handled and stored for the constitutional remit of EDTNA/ERCA only, and according to Swiss Law, EU regulations and national law of contracted service providers involved. Every person and entity involved has the right to get information from EDTNA/ERCA on personal data collected and managed by EDTNA/ERCA and its partners at any time and ask for a complete deletion.

<https://www.edtnaerca.org/resource/edtna/files/documents/general%20terms%20and%20conditions%20revised%20and%20amended%201.09.2020.pdf>

- **I hereby confirm I did read the text above and agree with rules of Data handling by EDTNA/ERCA**

**Are you happy to voluntarily participate in the survey?**

☐ Yes

☐ No

**Project leader**

…………………..RN, MSc, PhD(c)

EDTNA/ERCA Executive Committee Member & Publications Coordinator

**Survey Questionnaire**

**1**. **Country where you work**

…………….

**2. Gender**

□ Male

□ Female

**3. Age**

□ 21-30

□ 31-40

□ 41-50

□ 51-60

□ >60

**4. Years of working experience in general nursing**

□ 0-5

□ 6-10

□ 11-20

□ 21-30

□ 31-40

□ >40

**5. Years of working experience in nephrology nursing**

□ 0-5

□ 6-10

□ 11-20

□ 21-30

□ 31-40

□ >40

**6. Education level**

□ Bachelor Degree

□ Master Degree

□ PhD Degree

**7. Position at work**

☐ Staff Nurse

☐ Specialist Nurse

☐ Head Nurse /Nursing Manager

**8. Type of patients you attend to (please tick all that applies):**

☐ 1_HD patients in public hospital

☐ 2_HD patients in private centre setting

☐ 3_Home- HD/OLHDF patients attending outpatient clinic

**9. How many of your patients have officially been diagnosed with CKD-associated Pruritus (uremic pruritus, chronic itching)?**

☐ 0%-5%

☐ 6%-20%

☐ 21%-30%

☐ 31%-40%

☐ 41-50%

☐ over 50%

**10.** **Of those patients diagnosed with CKD-associated Pruritus, how many are mentioning / talking about their itching with you or your colleagues?**

☐ None

☐ 0%-5%

☐ 6%-20%

☐ 21%-30%

☐ 31%-40%

☐ 41-50%

☐ over 50%

**11. Of those diagnosed with CKD-associated Pruritus, what is their vascular access type:**

☐ A-V Fistula

☐ A-V Graft

☐ Central Venous Catheter

**12. Of those diagnosed with CKD-associated Pruritus, what is the method of their dialysis treatment:**

☐ 1_HD

☐ 2_OLHDF

☐ 3_Home Nocturnal HD/OLHDF

**13. Of those diagnosed with CKD-associated Pruritus, what is the dialyzer membrane type:**

☐ Low flux

☐ High Flux

☐ High Cut-off

**14. During the typical dialysis procedure, from the priming of blood lines & dialyzer until the moment the patient connects to and eventually disconnects from the dialysis session, are there any different nursing interventions you follow or additional steps that you take with the patients with CKD-associated Pruritus?**

☐ Yes

☐ No

**15. If yes, what are they (please tick all that apply)?**

☐ 1_special priming process- flushing with extra saline bloodlines & dialyzer

☐ 2_use of special dialyzer

☐ 3_administer tablets or IV antihistamine medication

☐ 4_administer tablets or IV corticosteroid medications

☐ 5_apply creams

**16. Do you think patients do not report CKD-associated Pruritus symptom?**

☐ Yes

☐ No

**17. If yes, why (please tick all that applies)?**

☐ 1_they don’t feel comfortable to talk about it

☐ 2_they feel embarrassed in case you see how bad their scratches are

☐ 3_they feel it is less important

☐ 4_they feel there is no available treatment for their problem

☐ 5_they don’t make the association between the symptom and the condition

**18. Do you ever prescribe yourself or recommend to the nephrologist and/or patient any treatments overall?**

☐ Yes

☐ No

**19. Do you ever prescribe yourself or recommend to the nephrologist and/or patient any treatment for itchiness specifically?**

☐ Yes

☐ No

**20. Are there any patients in your centre who are not officially diagnosed with pruritus and yet present symptoms of CKD-associated Pruritus?**

☐ Yes

☐ No

**21. If yes, can you estimate approximately what proportion of all your patients does that represent?**

☐ 0%-5%

☐ 6%-20%

☐ 21%-30%

☐ 31%-40%

☐ 41-50%

☐ over 50%

**22.** **In case you detect potential symptoms (namely itching) of CKD-associated Pruritus, do you document them in patient’s file?**

☐ Yes

☐ No

**23. In case you detect potential symptoms (namely itching) of CKD-associated Pruritus, who do you report them to (please tick all that applies)?**

☐ 1_nephrologist

☐ 2_other nurse colleagues

☐ 3_dermatologist

☐ 4_psychologist

☐ 5_family member

**24. Do you think that itching is considered by the nurses and other healthcare professionals as not linked to CKD?**

☐ Yes

☐ No

**25. Do you usually encourage the patient to discuss about the itching problems with other health professionals within the nephrology care team?**

☐ Yes

☐ No

**26. If yes, please specify who (please tick all that applies):**

☐ 1_nephrologist

☐ 2_other nurses

☐ 3_dermatologist

☐ 4_psychologist

☐ 5_family member

**27. Do you usually discuss a patient’s symptoms severity on CKD-associated Pruritus with the nephrologist?**

☐ Yes

☐ No

**28. If yes, what do you discuss about (please tick all that applies)?**

☐ 1_The impact CKD-associated Pruritus has on patient’s life

☐ 2_Possible medication options

☐ 3_The therapeutic results of medication administered during an HD session or medication taken at home

☐ 4_Other………………………………………………………………

**29. Do you take part in the decision-making process with regards to** **(please tick all that applies)?**

☐ 1_Increase / decrease the frequencies of dialysis session?

☐ 2_Increase / decrease in their duration?

☐ 3_Adjustments to the dialysis parameters

☐ 4_No, I am not involved in any way in the decision-making process for any of the above

**30. Do you ever discuss or interact with healthcare professionals other than the nephrologist with regards to a patient with CKD-associated Pruritus?**

☐ Yes

☐ No

**31. If yes, with who (please tick all that applies)?**

☐ 1_Dermatologist

☐ 2_Dietician

☐ 3_Pharmacist

☐ 4_Psychologist

☐ 5_Family doctor

**32. Do patients talk about the challenges that** **CKD-associated Pruritus can pose in the day-to-day life (chronic itching) with you?**

☐ Yes

☐ No

**33. If yes, what are these challenges (please tick all that applies)?**

☐ 1_sleep disorders

☐ 2_Social isolation

☐ 3_difficulties in physical / professional activities

☐ 4_Necessity to take additional medication to deal with itching or with new undesirable effects

☐ 5_Changes in their diet since the start of itching or other undesirable effects linked to it

☐ 6_Their feeling of embarrassment

☐ 7_Their feeling of depression

☐ 8_Sexual health issues

**34. Do you think patients don’t discuss CKD-associated Pruritus symptoms with their doctors?**

☐ Yes

☐ No

**35. If yes, why (please tick all that applies)?**

☐ 1_they don’t feel comfortable to talk about it

☐ 2_they feel embarrassed in case you see how bad their scratches are

☐ 3_they feel it is less important

☐ 4_they feel they will not get nursing/ medical attention on this issue

☐ 5_they don’t relate itchiness to CKD

**36. Do patients try self-medication for their CKD-associated Pruritus?**

☐ Yes

☐ No

**37. If yes, what do the try (please tick all that applies)?**

☐ 1_OTC creams

☐ 2_oils & herbs

☐ 3_antihistamine tablets

**38. How do they come up with those remedies (please tick all that applies)?**

☐ 1_Internet

☐ 2_Family member

☐ 3_Treating specialist

☐ 4_Suggested by another patient

☐ 5_I don’t know

**39. Are there any treatments specifically for the itching that you know of available for the patients with CKD-associated Pruritus (please tick all that applies)?**

☐ 1_Gabapentin/pregabalin

☐ 2_Antihistamines tablets

☐ 3_Antihistamines creams

☐ 4_Corticosteroids tablets

☐ 5_Corticosteroids creams

☐ 6_UVB (Ultraviolet phototherapy)

☐ 7_No available treatment

**40. Do relatives/ caregivers mention /discuss itchiness with you?**

☐ Yes

☐ No

**41. If yes, is itching a major topic of concern for the family?**

☐ Yes

☐ No

**Thank you for your valuable contribution in completing this survey**
